# Patient characteristics and utilization of an online patient portal in a rural academic general internal medicine practice

**DOI:** 10.1186/s12911-022-01778-w

**Published:** 2022-02-16

**Authors:** Stephen K. Liu, Annette E. Osborn, Sigall Bell, John N. Mecchella, Shoshana Hort, John A. Batsis

**Affiliations:** 1 Department of Medicine, Section of General Internal Medicine, Geisel School of Medicine at Dartmouth, Dartmouth-Hitchcock Medical Center, One Medical Center Drive, Lebanon, NH 03756-0001 USA; 2 Mount Holyoke College, South Hadley, MA USA; 3 Norwich, VT 05055 USA; 4 Department of Medicine, Beth Israel Deaconess Medical Center, Harvard Medical School, Boston, MA USA; 5 Department of Medicine, Section of Rheumatology, Geisel School of Medicine at Dartmouth, Dartmouth-Hitchcock Medical Center, One Medical Center Drive, Lebanon, NH 03756-0001 USA; 6 Division of Geriatric Medicine, School of Medicine and the Department of Nutrition, Gillings School of Global Public Health, University of North Carolina at Chapel Hill, CB# 7550, Chapel Hill, NC 27599-7550 USA

**Keywords:** Patient portal, Disparities, Rural, Health information

## Abstract

**Background:**

Online patient portals have the potential to improve patient engagement and health care outcomes. This is especially true among rural patient populations that may live far from their health care providers and for whom transportation is a barrier to accessing care. This study compared the characteristics of active users of an online patient portal to non-users and assessed utilization among users in a rural academic primary care clinic to identify disparities in adoption and use.

**Methods:**

We conducted a cross sectional study of 28,028 patients in a general internal medicine clinic between June 2019 and May 2020 to assess (a) characteristics of patients who had an online patient portal account and used the patient portal compared to those who did not register for an account, and (b) the frequency of use of the patient portal (number of logons and number of messages sent and received) by patients over the study period. We compared results based on demographic characteristics, focusing on gender, age, race, presence or absence of nine chronic illnesses, smoking status, and BMI.

**Results:**

In the study cohort of 28,028 patients, 82% were active users of the patient portal. Females, patients aged 41–65, and non-smokers were more likely to use the portal than their counterparts. In total, patients with eight out of nine chronic illness groups studied (heart failure, cerebrovascular disease, history of a myocardial infarction, peripheral vascular disease, and renal disease) were less likely to use the patient portal than patients without these chronic conditions. On average, patients log onto the patient portal 25 times per year and send and receive 6 messages to and from the clinic. We found that females, patients older than 65, former smokers and obese patients logged on and sent and received more messages compared to the overall cohort. Although the sample size was small, on average Black patients logged onto the patient portal 19 times and sent and received 3.6 messages compared to White patients who logged on 25 times with 5.8 messages on average over the yearlong study period.

**Conclusions:**

In a rural academic internal medicine clinic, female patients, aged 41–65, non-smokers, and those without certain chronic conditions were more likely to use an online patient portal. Recognizing and addressing barriers to patient portal use is essential for robust and sustained patient portal uptake and ensuring that the benefits of portal use are equally distributed among all patients.

## Background

Online patient portals provide the opportunity for patients to better understand and engage with their own health care and connect with their clinic and health care providers [1–3]. Utilizing common portal functionalities such as reviewing progress notes, visit summaries, and communicating with providers outside of traditional office visits may encourage patients to follow recommended care plans in order to better manage chronic health conditions and improve their health outcomes [2, 4, 5].

Despite the potential advantages, there remain many barriers to accessing online patient portals. Poor digital and health literacy, lack of internet access, unawareness of or forgetting about a patient portal’s existence, feeling inadequately trained to use the portal, and privacy concerns are all reported barriers to patient portal access [1, 4, 6, 7]. Black, Latinx, older patients and patients with chronic health conditions are known to have lower enrollment and use of an online patient portal [7–9].

In Northern New England, many rural patients have issues with access to health care providers and can often live far from their primary care clinic. Patients also may have difficulties with transportation to and from their health care clinic due to the distance and lack of public transportation. With increasing broadband and internet access over the past few years, many have turned to the patient portal to reduce their need for in-person clinic visits. Previously published studies about patient portal use have primarily examined urban (or mixed urban and rural) populations that may differ in terms of physical distance to their health care providers, access to health care providers, availability of internet access or had overall low rates of portal enrollment [7, 9–12].

The objective of this study was to identify characteristics of patient portal users compared to non-users and assess utilization through number of logons and portal messaging in an academic general internal medicine practice in rural New Hampshire with high rates of patient portal enrollment. Understanding utilization among a defined population of patients is important for planning for staffing needs to respond to and address the increasing volume of portal messages that are sent by patients through the patient portal.

## Methods

This cross-sectional study was conducted at Dartmouth-Hitchcock Medical Center, a rural academic medical center located in Lebanon, New Hampshire that serves a population of approximately 1.9 million patients from New Hampshire and Vermont [13]. All adult patients who were assigned to a primary care provider in the general internal medicine practice between June 2019 and May 2020 were included in this study. Primary care providers in the general internal medicine clinic included physicians, physician assistants and advanced practice nurse practitioners.

Patient data and portal use were obtained through a query from our data warehouse. The hospital system uses the EpicCare™ electronic health record for ambulatory care and the MyChart™ patient portal. Information recorded included demographic information (age, sex), clinical information (body mass index and chronic illnesses), and patient portal use over the study period. Active portal users had an account on the patient portal and logged onto the system during the study period. Message counts represented messages sent between the providers/clinic and patients and included messages sent from the clinic (which could include requests to complete surveys before upcoming appointments and reminders of upcoming appointments) and from patients to their providers during the study period.

Defined chronic illnesses were based on internal billing codes using International Classification of Disease, Tenth Edition (ICD-10). Identified chronic illnesses were selected from the Charlson Comorbidity Index and excluded conditions for which only a small number of patients had the condition such as paraplegia, rheumatic disease, HIV, and liver disease [14]. They were dichotomized (present/absent) based on the established groupings of codes by condition. A patient was noted to have a specific chronic illness if they had the specified condition listed in their problem list or a visit diagnosis from one of the disease groupings was included in a clinic visit during the study time period. Defined chronic illnesses included congestive heart failure, chronic obstructive pulmonary disease (COPD), cerebrovascular disease, diabetes mellitus with and without complications, history of a myocardial infarction (MI), peripheral vascular disease, and renal disease.

### Statistical analysis

After data extraction, we conducted internal data validity checks and imported the data for further analyses. Descriptive statistics were evaluated on the entire cohort. We then stratified our cohort by active users of the portal and non-active users to evaluate the differences in baseline characteristics that could potentially influence use. We conducted unpaired t-tests of unequal variance or chi-squares (or their non-parametric equivalences) comparing portal and non-portal users. The null hypothesis was that portal users were younger, more often of white race, more often never smokers, and had lower prevalence rates of chronic medical illness. Further, we hypothesized that healthier weight (e.g., normal BMI) rather than underweight, overweight or obesity was associated with higher portal use. All descriptive statistics were two-sided, and *P*-values < 0.05 were considered statistically significant. All analyses were performed using STATA v.15 (College Station, TX).

The study was reviewed and approved by the Dartmouth-Hitchcock Institutional Review Board (Study #00,006,738) and was considered a minimal risk study and did not require review by the institutional Ethics Committee.

## Results

### General characteristics of portal users

A total of 28,028 patients in the general internal medicine clinic were evaluated during the study period. Among these, 22,955 (82%) of patients were active portal users (Table 1). Patients who were female, aged 41–65, and non-smokers were more likely to be active users of the portal while current smokers were significantly less likely to use the portal.

**Table 1:** Overall characteristics of study patients in the overall cohort and active and non-users of the patient portal

	Overall cohort N = 28,028	Active users of portal	Non-users of portal	*p* value
Number of patients	28,028	22,955 (81.9%)	5071 (18.1%)	
Female	16,138	13,684 (84.8%)	2453 (15.2%)	*P* < 0.001
Age, years				*P* < 0.001
18–40	6021	5006 (83.1%)	1015 (16.9%)	
41–65	11,425	9605 (84.1%)	1819 (15.9%)	
> 65	10,582	8344 (78.9%)	2237 (21.1%)	
Race				*P* < 0.001
White	26,706	21,863 (81.9%)	4842 (18.1%)	
Black	220	161 (73.2%)	59 (26.8%)	
Asian/Pacific Islander	556	485 (87.2%)	71 (12.8%)	
Native American	86	65 (75.6%)	21 (24.4%)	
Other	460	381 (83.0%)	78 (17.0%)	
Smoking status				*P* < 0.001
Never	15,545	13,271 (85.4%)	2274 (14.6%)	
Former	9262	7643 (82.5%)	1619 (17.5%)	
Current	2613	1714 (66.0%)	899 (34.0%)	
Unknown	608	327 (54.0%)	279 (46.0%)	
Chronic illness				
Congestive heart failure	1833	1246 (68.0%)	587 (32.0%)	*P* < 0.001
COPD	7805	6329 (81.0%)	1476 (19.0%)	*P* = 0.03
Cancer	3789	3104 (81.9%)	685 (18.1%)	*P* = 0.98
Cerebrovascular disease	2670	1965 (73.6%)	705 (26.4%)	*P* < 0.001
Diabetes mellitus with complications	1923	1395 (72.5%)	528 (27.5%)	*P* < 0.001
Diabetes mellitus without complications	4218	3192 (75.7%)	1025 (24.3%)	*P* < 0.001
History of MI	1486	968 (65.1%)	518 (34.9%)	*P* < 0.001
Peripheral vascular disease	4117	2998 (72.8%)	1119 (27.2%)	*P* < 0.001
Renal disease	1950	1441 (73.9%)	509 (26.1%)	*P* < 0.001
BMI category				*P* < 0.001
Underweight (< 18.5)	396	287 (72.5%)	109 (27.5%)	
Normal (18.5–24.9)	8170	6906 (84.5%)	1264 (15.5%)	
Overweight (25.0–29.9)	8724	7281 (83.5%)	1443 (16.5%)	
Obese (≥ 30.0)	9846	7954 (80.8%)	1892(19.2%)	
Unknown	892	527 (59.1%)	365 (40.9%)	

*COPD* chronic obstructive pulmonary disease, *MI* Myocardial Infarction, *BMI* Body Mass Index

Of all chronic illnesses evaluated, eight out of nine demonstrated lower portal use compared to the overall cohort: Patients with heart failure, cerebrovascular disease, diabetes mellitus, history of MI, peripheral vascular disease and renal disease were significantly less likely to use the portal.

We also examined BMI categories and patients who were classified as being underweight (BMI < 18.5) or having obesity (BMI ≥ 30.0) were the least likely to be active users of the portal compared to normal (BMI 18.5–24.9) or overweight patients (BMI 25.0–29.9).

### Frequency of portal use

For the overall cohort, on average, a patient logged onto the patient portal 25 times and sent and received approximately 6 messages over the one-year study period (Table 2). We found that females, patients older than 65, former smokers and obese patients (BMI ≥ 30.0) logged on and sent and received more messages compared to the overall cohort. While the examined minority population was small (220 Black patients and 556 Asian/Pacific Islander patients) in our study, on average Black patients logged on 19 times with 3.6 messages sent and received over the study period and Asian patients logged on 17.9 times with 2.7 messages compared to White patients who logged on 25 times with 5.8 messages per year (Table 2).

**Table 2:** Average number of logons and messages sent and received during the one year study period in total study population

	Average logons in one year per patient (average ± SD) (maximum)	*P*-value	Average number of messages communicated in one year (average ± SD)	*P*-value
Overall	25.1 ± 47.0		5.8 ± 17.5	
Gender		*P* < 0.001		*P* < 0.001
Female	28.5 ± 48.1		6.7 ± 19.2	
Male	20.6 ± 45.1		4.5 ± 14.7	
Age		*P* = 0.76		*P* < 0.001
18–40	23.1 ± 47.6		4.2 ± 15.7	
41–65	25.2 ± 48.4		6.1 ± 19.5	
> 65	26.2 ± 45.1		6.3 ± 16.0	
Race		*P* < 0.001		*P* < 0.001
White	25.4 ± 47.2		5.8 ± 17.5	
Black	18.8 ± 37.0		3.6 ± 11.0	
Asian/Pacific Islander	17.9 ± 28.7		2.7 ± 7.8	
Native American	34.9 ± 81.2		10.5 ± 31.1	
Other	22.0 ± 50.4		5.5 ± 22.3	
Smoking status		*P* < 0.001		*P* < 0.001
Never	24.8 ± 45.7		5.4 ± 17.5	
Former	29.2 ± 51.0		7.1 ± 18.0	
Current	18.4 ± 43.0		4.8 ± 17.0	
Unknown	2.0 ± 5.6		0.1 ± 0.35	
BMI category		*P* < 0.001		*P* < 0.001
Underweight (< 18.5)	25.6 ± 48.1		5.8 ± 15.2	
Normal (18.5–24.9)	23.0 ± 41.1		5.0 ± 14.6	
Overweight (25.0–29.9)	24.4 ± 47.7		5.3 ± 16.3	
Obese (≥ 30.0)	29.5 ± 52.0		7.3 ± 21.1	
Unknown	2.62 ± 10.0		0.35 ± 3.1	

*BMI* Body Mass Index

## Discussion

In our rural academic general internal medicine clinic, we found an overall high uptake in patient portal use with 82% active users who regularly logged on and interacted with the clinic providers using the messaging function through the portal. The high rates of utilization demonstrated that rural patients are seeking out the patient portal to communicate to their health care providers and are able to access online resources with expansion of smart phone use and cellular and high speed internet availability throughout the region [15, 16]. Rural patients often have to travel significant distances to see their providers and an online platform enabling communication with the clinic and their health care providers can be a valuable tool to improve health outcomes outside of traditional office visits.

Importantly, we identified disparities in patient portal use with older patients, active smokers, underweight and obese patients, and the great majority of patients with examined chronic conditions demonstrating lower rates of portal utilization. A systematic review also found that multiple studies have shown lower use by racial and ethnic minorities but unlike the findings of our study, they also identified studies that showed greater use with increased numbers of medical problems [8].

We believe that older patients and those with multiple chronic diseases (including obesity) could be the patients who could benefit the most from use of a patient portal. These patients often have multiple specialists, medications and treatment plans and the ability to access their progress notes, review clinic visit summaries and messaging their providers with questions or health updates has the potential to improve their understanding of their treatment plans, communication with their health care team, patient engagement and their health outcomes. A key element of the Chronic Care Model is self-management support and a well-designed clinical practice could use patient portal messaging to educate patients and identify barriers in managing their chronic illnesses [17]. Unfortunately, we were not able to survey or interview patients with chronic diseases for this research project to identify the underlying reasons for the lower utilization. However, we speculate that the lower utilization rates are likely due to many different and interrelated contributors including education and income levels, access to technology, engagement with and trust in their health care team and knowledge about use of the patient portal. Clarifying these important contributors for lack of use is an important area for future research.

This study was also unique in that it examined a population of patients with a significantly higher percentage in patient portal use compared to previously published studies that may reflect increasing availability of high speed internet, cellular service and smart phone use since the publication of the previous studies [1, 9, 18]. The high rates of enrollment and use may also be a result of the clinic and providers’ encouragement and recommendations for portal use. Our institution has had an online patient portal for 20 years which was transitioned to the EpicCare™ electronic health record in 2011. The institution has actively encouraged patients to enroll in the patient portal and keeps them engaged with pre-visit questionnaires, messaging and prescription management. Providers within the clinic also encourage patients to enroll in the portal to review their progress notes and lab and radiology results and instructions on how to activate their account are included in every printed after visit summary for patients who have not activated their account.

This study was also able to quantify the volume of messages sent and received by patients which is important to quantify for appropriate clinic staffing. Over the one year study period, there were 137,730 patient portal messages sent and received by the clinic from the 22,955 active portal users. Unfortunately, our data was not able to be broken down into the percentage of messages that were initiated by patients and those that were sent by the clinic but as most of the messages sent by the clinic are in response to patient initiated messaging, both are important to characterize as they both require time and resources to address the patient portal messages. As patient portal use increases in use and begins to replace traditional face-to-face clinic visits and patient telephone calls, primary care clinics need to adapt their clinic staffing models to adequately address the needs of patient portal messaging.

Limitations of this study include that this was a single site study and a lack of data on social determinants of health such as patient education, income, digital literacy, or access to the internet or cellular service at their residence. All of these factors may significantly impact online patient portal use but unfortunately these data are not routinely available through data extraction from the electronic health record. While we were able to identify differences in patient portal use, we were not able to interview patients to identify causes or barriers that would help explain the observed differences in this study. Additionally, we were not able to validate data about race and confirm the reliability of documentation of chronic conditions with patients as the data that we obtained for this study was de-identified and the accuracy of problem lists and visit diagnoses could not be verified. Further research with focus groups and individual patient interviews would be very helpful to identify potential social determinants of health that are associated with decreased patient portal usage and to clarify barriers to accessing the patient portal in our rural region. More research should also be performed to evaluate the clinical outcomes of patients who utilize the patient portal and investigate whether use of the patient portal has an impact on rates of hospitalizations, emergency room visits, patient satisfaction, and other disease specific outcomes for diabetes or hypertension management. We also had a very small number of minority patients in our study population and additional studies in rural centers with a more diverse patient population are needed to identify racial and economic factors that prevent utilization of the patient portal.

## Conclusion

In a rural academic general internal medicine clinic, we found high patient portal usage overall but lower rates among males, active smokers, older patients and those with examined chronic illnesses. Recognizing and addressing barriers to patient portal use is essential for robust and sustained patient portal uptake and ensuring that the benefits of portal use are equally distributed across the population of all patients.

## Data Availability

The datasets analyzed during the current study are not publicly available due to institutional privacy and data sharing policies but a de-identified data set without any personal health information is available from the corresponding author on reasonable request if approval is granted by the institutional privacy office through a data use agreement and through the Institutional Review Board.
